# Fibroin-Hybrid Systems: Current Advances in Biomedical Applications

**DOI:** 10.3390/molecules30020328

**Published:** 2025-01-15

**Authors:** Matheus Valentin Maia, Eryvaldo Sócrates Tabosa do Egito, Anne Sapin-Minet, Daniel Bragança Viana, Ashok Kakkar, Daniel Crístian Ferreira Soares

**Affiliations:** 1 Laboratório de Bioengenharia, Universidade Federal de Itajubá, Itabira 35903-087, Minas Gerais, Brazil; matheus.maia@mail.mcgill.cadanieltumbling@unifei.edu.br (D.B.V.); 2 Department of Chemistry, McGill University, 801 Sherbrooke Street West, Montréal, QC H3A 0B8, Canada; 3 Laboratório de Sistemas Dispersos LaSiD, Faculdade de Farmácia, Universidade Federal do Rio Grande no Norte, Natal 59012-570, Rio Grande do Norte, Brazil; socratesegito@gmail.com; 4 Faculté de Pharmacie, Université de Lorraine, CITHEFOR, F-54000 Nancy, France; anne.sapin@univ-lorraine.fr

**Keywords:** silk fibroin, hybrid system, surface-grafting polymerization, chemical modification, biomedical applications

## Abstract

Fibroin, a protein extracted from silk, offers advantageous properties such as non-immunogenicity, biocompatibility, and ease of surface modification, which have been widely utilized for a variety of biomedical applications. However, in vivo studies have revealed critical challenges, including rapid enzymatic degradation and limited stability. To widen the scope of this natural biomacromolecule, the grafting of polymers onto the protein surface has been advanced as a platform to enhance protein stability and develop smart conjugates. This review article brings into focus applications of fibroin-hybrid systems prepared using chemical modification of the protein with polymers and inorganic compounds. A selection of recent preclinical evaluations of these hybrids is included to highlight the significance of this approach.

## 1. Introduction

Silk is a natural, protein-based material produced by arthropods, including arachnids and silkworms, such as the Bombyx mori species. Silkworms, not domesticated silk moths, are the producers of silk, which has been used by humans for thousands of years due to its luster, mechanical strength, and flexibility. These properties arise from their unique chemical and structural interactions across hierarchical length scales [1,2,3]. Silk threads are composed of two fibroin brins coated with sericin, glycoproteins, and lipids. Silk fibroin (SF) from Bombyx mori consists of a 325 kDa heavy chain linked to a 25 kDa light chain through a disulfide bond. While the heavy chain is primarily composed of repetitive amino acids such as alanine, glycine, and serine, the amino acid composition of the light chain is more variable and lacks the same repetitive motifs [1,2]. Biodegradability, biocompatibility, and non-immunogenicity of silk-based materials have attracted considerable interest, especially for biomedical applications [3,4]. Fibroin, a protein extracted from silk, has emerged as a highly promising material in this regard, primarily due to its remarkable physicochemical and biological properties, which include its ability to encapsulate various therapeutic agents, such as proteins, enzymes, genetic materials (e.g., DNA, RNA), and anticancer drugs, making it a versatile platform for drug delivery [5,6].

Fibroin-based nanoparticles and hydrogels allow precise control over particle size and drug release profiles, facilitating controlled and sustained release of therapeutic molecules at the target site [5,6,7]. Fibroin has shown exceptional potential in tissue engineering, where it can serve as a scaffold for regenerating tissues, including skin, bone, cartilage, and even nerve tissues, due to its high mechanical strength, biocompatibility, and ability to support cell adhesion and proliferation [8,9,10]. Fibroin has also been explored in wound healing, where its hemostatic properties and ability to facilitate tissue regeneration promote rapid recovery [11,12]. Recent studies have demonstrated the use of fibroin in advanced biomedical applications, such as the development of bioinks for 3D bioprinting, biosensors for diagnostic purposes, and antimicrobial coatings for medical devices [13,14]. These versatile applications underscore the adaptability and importance of fibroin in modern biomedical research and clinical practice.

**Figure 1:**
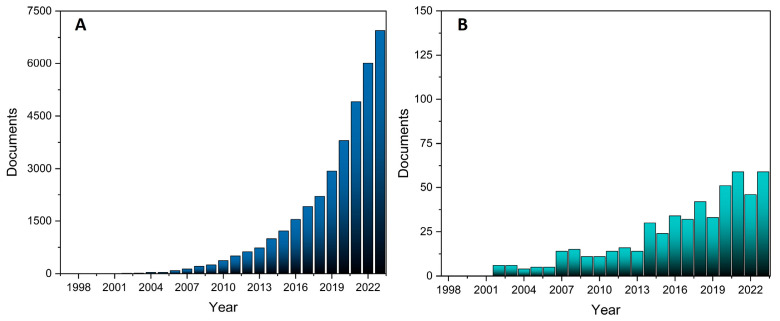
Publications search analysis based on the Scopus^®^ database records using the keywords: fibroin nanoparticles (**A**) and fibroin hybrid (**B**).

Despite its numerous advantages, fibroin presents several challenges that can hinder its efficacy in biomedical applications. One of the most significant limitations is its susceptibility to enzymatic degradation by proteases in vivo, which compromises its structural and functional stability in biological environments. This degradation reduces its efficacy as a material for drug delivery systems or structural scaffolds in tissue engineering [10,15,16,17]. Furthermore, fibroin’s instability under physiological conditions often results in inconsistent performance, necessitating the use of stabilizing agents or chemical modifications to enhance its durability and functionality [17,18,19]. To address the above-mentioned limitations, recent research has focused on advanced chemical modification techniques [15,16,17,18,19]. A key strategy involves the functionalization of fibroin with polymers to enhance its stability and solubility under physiological conditions. Polymer functionalization not only increases fibroin’s resistance to enzymatic degradation but also improves its capacity for controlled drug release, thereby extending its therapeutic efficacy, for example [15,16,20,21]. Additionally, inorganic compounds such as hydroxyapatite and silica have been conjugated into fibroin matrices to form hybrid systems, which could offer new venues for applications [22,23,24]. Studies on developing fibroin-hybrid nanoparticles are relatively recent, and from the data obtained from Scopus using the search keyword “fibroin nanoparticles”, it is noted that only a few papers were published annually until the year 2007 (Figure 1A). However, significant growth has been observed since 2014, and a 5000-document milestone was reached in 2021 (Figure 1A), and in 2023, a grand total of 6945 articles were published. In Figure 1B, publications reported specifically using the keyword “fibroin hybrid” are shown, which indicates a steady increase in studies over the past two decades. This suggests that the development and application of hybrid systems is becoming a major focus in recent years.

Fibroin has a significant number of amino acids located in its side chains with functional groups such as amine, sulfhydryl, and carboxyl groups, which are sites at which chemical modification can be efficiently conducted, leading to the formation of hybrid derivatives or composites. Furthermore, such modifications can add other relevant characteristics to the protein, highlighting thermal, photo, and redox responsiveness [25,26,27,28]. For example, fibroin has been associated with gold and silver metals aiming to produce hybrid materials for antibacterial and sensing devices [29,30]. In addition, the protein has been linked to metal oxides of iron and zinc for tissue engineering applications [31,32]. Biodistribution studies have revealed relevant in vivo characteristics of the hybrid fibroin nanoparticles, which can accumulate in tumor regions, significantly reducing the growth rhythm of cancer cells, displaying prolonged blood circulation time, reducing hemolysis, and accumulating in non-target tissues such as the heart, spleen, liver, and lungs [33,34,35].

**Figure 2:**
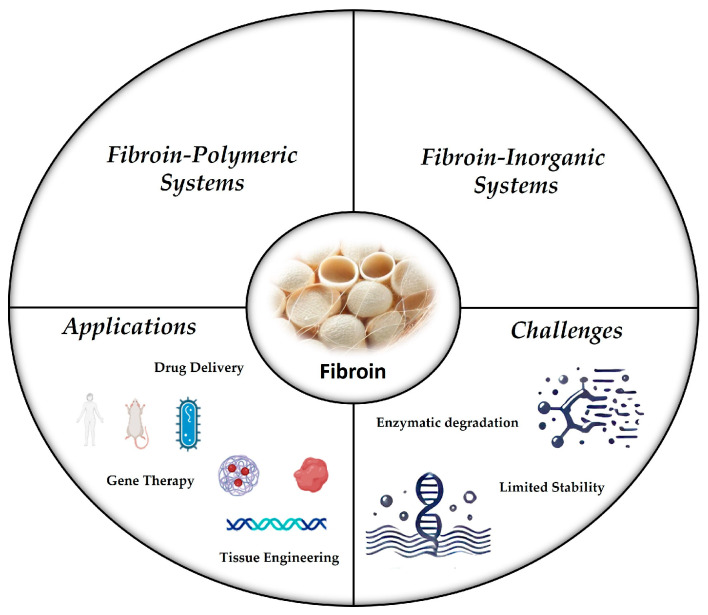
Schematic representation of fibroin-based hybrid systems with emphasis on two main categories: polymer- and inorganic-hybrid systems. Key applications include tissue engineering, drug delivery, and gene therapy, with challenges such as enzymatic degradation and limited stability.

This review brings into focus the most recent advances in fibroin functionalization, specifically focusing on the grafting of polymers and inorganic compounds onto the protein surface (Figure 2). Regarding studies involving polymers and fibroin, only reports that feature chemical modifications are discussed, excluding those that rely solely on physical mixing techniques such as electrospinning. It also aligns with the IUPAC definition of hybrid materials. Additionally, this review highlights advanced applications of fibroin-based hybrid systems and a selection of recent preclinical studies, which are included to show the importance and potential impact of this approach in advancing biomedical applications.

## 2. Fibroin: Isolation and Purification

The importance of obtaining pure protein cannot be overstated when aiming to perform chemical modifications on fibroin. Impurities, such as sericin and other residual components, can interfere with functionalization and compromise the intended properties of the modified material. High-purity fibroin ensures that its functional groups, such as hydroxyl, carboxyl, and amine, remain accessible and reactive during modification steps. Furthermore, the removal of impurities prevents unwanted side reactions that could negatively affect the mechanical, thermal, and biological properties of the resulting hybrid systems. Purity is also crucial for the reproducibility of chemical reactions, allowing for the precise tailoring of fibroin-based materials for specific applications.

**Figure 3:**
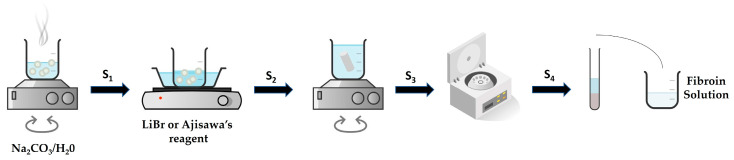
General procedure for fibroin isolation. The raw silk cocoons are boiled in a solution of sodium carbonate to remove sericin (S_1_), and the silk fibers are dissolved in a highly concentrated solution, such as lithium bromide or Ajisawa’s reagent, to break down the fibers into a fibroin solution (S_2_). The fibroin solution is dialyzed against distilled water to remove the solvent and any other small molecules (S_3_), resulting in a purified fibroin solution, and at the end of the process, the solution is centrifuged (S_4_) to remove any remaining impurities.

To achieve acceptable levels of purity, fibroin extraction involves a selective and rigorous process (Figure 3, Table 1), typically beginning with degumming (Figure 3, S_1_) using an alkaline treatment, which is the most widely used method for degumming fibroin from Bombyx mori silk cocoons [36]. The degumming protocol involves cutting silk cocoons into small pieces and boiling them in a 0.02 M aqueous sodium carbonate (Na₂CO₃) solution for about 30 to 35 min under constant agitation. Sodium carbonate selectively solubilizes sericin that coats fibroin threads, while fibroin itself remains insoluble, forming a spongy mass. This mass is then thoroughly washed with deionized water and dried overnight at room temperature [36,37,38].

Once fibroin is dried, solubilization (Figure 3, S_2_) can be carried out using a variety of methods. Popular approaches include the addition of lithium bromide (LiBr) [39,40,41] or Ajisawa’s reagent (CaCl₂/EtOH/H₂O) [42,43,44]. Ajisawa’s reagent is particularly favored due to its cost-effectiveness and efficiency. After solubilization, the fibroin solution undergoes purification through dialysis (Figure 3, S_3_) against distilled water for 72 h, which removes low-molecular-weight impurities. Following dialysis, the solution is centrifuged (Figure 3, S_4_) to separate any remaining impurities, leaving a purified fibroin in the supernatant. Table 1 summarizes important steps and possible different methods in the isolation and purification of fibroin, together with varied pitfalls in each.

The resulting fibroin solution, free of contaminants, can then be stored under refrigeration for future applications or lyophilized. This pure fibroin solution is critical for successful chemical modifications, such as polymer grafting or the incorporation of inorganic compounds, which can significantly enhance its properties for applications in biomedical engineering and material science.

Table 1 summarizes the steps involved in the purification of fibroin, with details on varied methods employed in each step, including details of chemical reagents that are utilized, the purpose of each, and drawbacks that are associated with these.

Despite the numerous advantages that silk fibroin offers in biomedical applications, including biocompatibility, biodegradability, and mechanical properties, the use of pure fibroin presents several disadvantages that limit its direct use [6,45]. One of the main limitations is the difficulty in controlling its degradation rate. Fibroin degradation can be unpredictable, depending on the biological environment and the conditions of the tissue where it is implanted [45,46,47,48]. This variability can result in an uncontrolled release of any incorporated therapeutic agent, compromising treatment efficacy. Another significant disadvantage is the lack of specific functionality in pure fibroin. Although fibroin has favorable mechanical properties, it does not possess functional groups that allow easy chemical modification or binding of bioactive molecules such as growth factors, antibiotics, or other therapeutic proteins, which poses a problem in applications that require specific biochemical interactions with cells or tissues [49,50].

Given these limitations, there is an unmet need to develop hybrid materials that combine silk fibroin with other materials. Protein-polymer hybrid materials have the potential to overcome several disadvantages of using pure fibroin. For example, the incorporation of polymers can improve biodegradation, mechanical stability, and surface functionality of fibroin-based materials [21,51,52]. Modifying fibroin with polymers can introduce additional functional groups that facilitate the binding of bioactive molecules, allowing the synthesis of more advanced medical devices, such as scaffolds for tissue engineering or controlled drug release. This approach allows tailoring materials more precisely for different medical applications, matching them with each application’s unique requirements [6,45,50,52,53,54]. Therefore, the development of protein-polymer hybrid materials not only mitigates the disadvantages of pure fibroin but also significantly expands its potential use in regenerative medicine, drug delivery systems, and other advanced medical devices.

**Table 1:** Summary of Steps, Methods, and Drawbacks in Fibroin Purification.

Process	Method	Reagent/Condition	Purpose	Drawbacks	Reference
Degumming (S_1_)	Alkaline treatment	Sodium carbonate (0.02–0.05 M), 90–100 °C	Removes sericin to expose fibroin	Harsh conditions can degrade fibroin, reducing its molecular weight and affecting its properties.	[6,36]
	Enzymatic treatment	Protease enzymes (e.g., papain)	Selective sericin removal	Costly and time-consuming; requires precise pH and temperature control.	[55,56]
	Soap-based treatment	Mild soap solution, 90–100 °C	Gentle removal of sericin	Incomplete sericin removal can occur, affecting fibroin purity.	[57,58]
Solubilization (S_2_)	Chemical dissolution	Lithium bromide (9.3 M), 60 °C	Dissolves fibroin into solution	High salt concentration requires extensive dialysis;	[36]
	Ionic liquid dissolution	Ionic liquids (e.g., 1-ethyl-3-methylimidazolium acetate)	Dissolves fibroin with minimal degradation	Expensive reagents; recovery and reuse of ionic liquids are challenging.	[59,60]
	Acidic dissolution	CaCl₂-ethanol-water system, 60 °C	Dissolves fibroin for further processing	Corrosive reagents may damage equipment; fibroin structure can be altered.	[42,43,44]
Purification (S_3_)	Dialysis	Distilled water	Removes salts, chemicals, and impurities	Time-intensive process; incomplete removal can leave residual salts.	[6,36]
Filtration (S_4_)	Centrifugation	High-speed centrifuge	Separates insoluble particles and aggregates	Requires specialized equipment; energy-intensive.	[6,36]

## 3. Polymer-Fibroin Hybrid Materials

Considering scientific resources, in particular SCOPUS and Web of Science, the two largest and most complete databases of abstracts and citations, it is possible to find several publications that present in their title, keywords, or abstract words such as “hybrid systems” or “hybrid nanostructures”. However, it can lead to misunderstanding of what, in fact, the term hybrid system means. According to IUPAC (International Union of Pure and Applied Chemistry), hybrid materials are composed of an intimate mixture of inorganic and/or organic components, which usually interpenetrate on a scale of less than 1 μm [61], and are the result of atomic-level interactions in multicomponent interfaces that result in materials with synergistic final properties of their components, including the presence of a very large hybrid interface [62]. In this context, composites and blends are not classified as hybrid materials. Blends constitute a mixture of two or more materials, potentially incompatible, without forming a new chemical entity. This involves a mere physical combination of materials, resulting in properties that are typically an average of the individual components. Conversely, composites entail the merging of distinct materials to generate a novel material with improved characteristics. In composites, components are frequently integrated at a macroscopic level, wherein one material serves as a matrix, and the others act as reinforcements.

The most common hybrid materials are organic–inorganic [63,64,65], but organic-organic hybrid materials have also played an important role in different fields, especially in biomedical applications [66,67]. Furthermore, the conjugation of synthetic polymers with various biomolecules, such as proteins, polysaccharides, and many others, combines the advantages of both natural and synthetic polymers [68]. Since 1977, when Abuchowski et al. [69] and later Kanamaru et al. [70] reported that PEGylated proteins increased circulation times in animal models relative to native proteins, there has been a significant increase in studies related to protein-polymer conjugates for therapeutic applications. Important advances have been made in the synthesis of hybrid nanomaterials that provide promising alternatives to conventional modalities and have increased the possibility of applications in biomedical areas. More specifically, the reversible-deactivation radical polymerization (RDRP) methodologies are currently the most promising routes to prepare conjugates with well-defined polymer molecular weights [6,52]. Grafting methods based on RDRP, such as atom transfer radical polymerization (ATRP) or reversible addition-fragmentation chain transfer (RAFT), show superiority over other methods for the synthesis of new hybrid systems, as they can provide a higher degree of control over the polymerization process, resulting in more precise and complex polymer structures with tailored properties. The latter is often essential for advanced applications in materials science, medicine, and nanotechnology [66,71,72,73].

In recent years, an increasing number of SF-based hybrid systems have been used for applications including incorporation and controlled release of drugs, tissue engineering and regenerative medicine, disease models, implantable devices, and others [74,75,76]. Although several promising studies using only fibroin in medical applications are widely found in the literature, there is an increasing trend to optimize and equip these structured nanosystems with specific functions. Synthesizing a hybrid system based on fibroin and organic polymers can be considered a promising approach to obtain nanosystems with unique properties. Combining different characteristics, the systems based on SF and polymers can allow several advantages over non-hybrid materials, such as the optimization of circulation time in the bloodstream, a high encapsulation rate, and specific release kinetics for drug delivery systems. In addition, in the tissue engineering area, these materials can improve mechanical properties and allow fine control of tissue stimulation [66,77,78,79]. A survey of the SF-polymer hybrid shows significant advancements in biomaterial development, and Table 2 summarizes the key hybrid systems, their physicochemical properties, biomedical applications, and characterization methods while highlighting their potential for innovative therapeutic applications.

Buga et al. [52] explored surface modification of SF fibers by grafting poly(methyl methacrylate) (PMMA) and poly(tributylsilyl methacrylate) (PTBSiMA) using RAFT polymerization. The fiber surfaces were functionalized with a silane coupling agent to introduce reactive vinyl groups. These groups facilitated the subsequent polymerization of MMA and TBSiMA, creating polymer chains chemically bound to the fibroin surface. Characterization confirmed successful grafting, with modified fibers showing improved thermal stability and well-defined morphologies. The inclusion of PTBSiMA, with its hydrolyzable silyl ester groups, contributed to erosion resistance and self-renewing surface properties, particularly in aqueous environments. These attributes highlight the potential for antifouling applications, traditionally used in marine contexts, to be extended into biomedical fields. In biomedical applications, antifouling properties are critical for preventing protein adsorption, bacterial adhesion, and biofilm formation on device surfaces. The self-renewing and non-fouling characteristics of the PTBSiMA-modified fibroin fibers could make them suitable for medical devices such as catheters, implants, and biosensors, where maintaining clean and biocompatible surfaces is essential.

Considering intriguing properties and potential applications of fibroin in the biomedical area, Xing et al. [80] explored the development of a polymer hybrid material by modifying the surface of SF using atom ATRP. It involved grafting dimethylaminoethyl methacrylate (DMAEMA) onto the fibroin surface, followed by polymerization, to generate a material with antibacterial properties. The process began with the preparation of a macroinitiator by functionalizing the amino and hydroxyl groups on silk fibroin using 2-bromoisobutyryl bromide (BriB-Br). The resulting SF-Br macroinitiator served as a foundation for ATRP, which polymerized DMAEMA in the presence of copper bromide (CuBr) and a ligand, N′′,N′′-pentamethyldiethylenetriamine (PMDETA), in dichlorobenzene. It led to a smooth, uniform polymer layer on the fibroin surface without any unwanted homopolymers. Following polymerization, the tertiary amine groups (in DMAEMA) were reacted with methyl bromide, introducing quaternary ammonium functionalities that enabled the material with potential antibacterial properties. Characterization using techniques including Fourier transform infrared spectroscopy (FT-IR) and X-ray energy dispersive spectroscopy (EDS) was utilized to confirm successful grafting of DMAEMA and the presence of bromine on the fibroin surface. Scanning electron microscopy (SEM) revealed a smooth polymer coating, while gel permeation chromatography (GPC) demonstrated low polydispersity and controlled molecular weights of the grafted polymers. The authors evaluated the antibacterial properties of silk fibroin grafted with quaternary ammonium groups onto the hybrid’s surface. These groups are known for their antibacterial activity due to their ability to disrupt microbial membranes. Although this study does not provide specific experimental details about the antibacterial assays, it highlights that the quaternized surface was designed to exhibit antibacterial potential. Typically, such evaluations would involve testing against common bacterial strains, such as Staphylococcus aureus or Escherichia coli, using assays like zone of inhibition, colony counting, or measuring bacterial growth in contact with the material. The quaternization process ensures a high concentration of quaternary ammonium groups, which likely contributes to the enhanced antibacterial performance of the silk fibroin material. Further evaluations of these systems, including specifics about the bacterial strains, incubation conditions, and quantitative or qualitative assessment methods used to measure antibacterial efficacy, are needed.

In 2019, Radu et al. [81] developed a hybrid system with silk fibroin grafted with poly(N-isopropylacrylamide) (SF-g-PNIPAM), synthesized through radical polymerization initiated by cerium ammonium nitrate (CAN). By grafting PNIPAM, a thermosensitive polymer, onto silk fibroin, the material gained responsiveness while maintaining biocompatibility and mechanical properties inherent to fibroin. This hybrid material demonstrated significant potential for advanced biomedical applications, particularly in drug delivery and responsive biomaterials. Grafting was achieved by initiating radical polymerization at the tyrosine residues of silk fibroin, facilitated by the cerium-based redox system. The resulting SF-g-PNIPAM was characterized to confirm successful grafting and to understand its structural and functional changes. FTIR and Raman spectroscopies confirmed the introduction of PNIPAM onto the fibroin backbone, while XPS analysis verified chemical modifications. Thermal analysis showed increased thermal stability, and CD and XRD analyses revealed structural rearrangements, with a decrease in β-sheet content and alterations in crystallinity. SEM imaging highlighted changes in surface morphology, indicating successful integration of PNIPAM. The most notable applications of this hybrid material were demonstrated through its potential in smart drug delivery systems. The thermosensitive nature of PNIPAM enabled controlled release of hydrophilic drugs, such as 5-fluorouracil (5-FU), through controlled responses to temperature changes. Drug release assays showed that the SF-g-PNIPAM hybrid could achieve sustained and targeted delivery, optimizing therapeutic efficacy. Additionally, the hybrid material’s biocompatibility and structural integrity make it suitable for use in hydrogels, which are scaffolds for tissue engineering and wound healing systems. The combination of mechanical stability and temperature responsiveness creates an ideal platform for applications requiring precision and adaptability, such as regenerative medicine and responsive therapeutic systems. Overall, the results emphasized the hybrid material’s ability to enhance biomedical applications by combining the intrinsic properties of silk fibroin with the tunable functionalities of PNIPAM.

**Figure 4:**
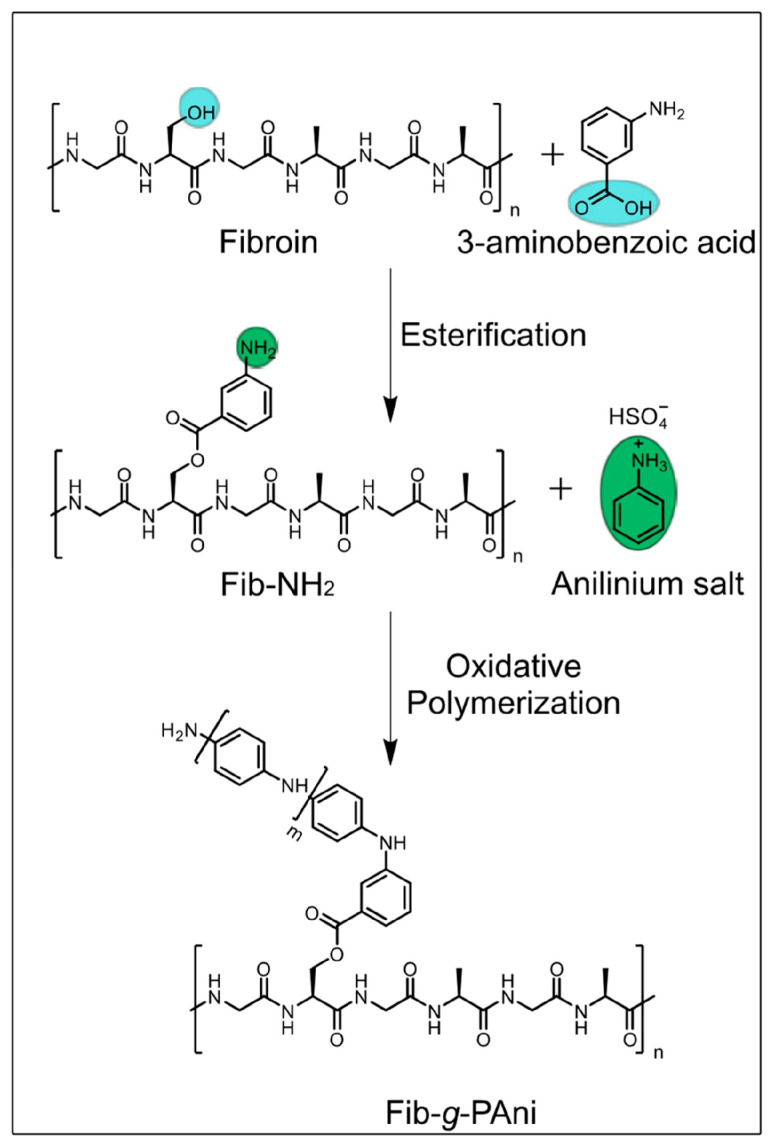
Scheme for the synthesis of fibroin-polyaniline (PAni) hybrid. Reproduced and adapted with permission from [82].

**Figure 5:**
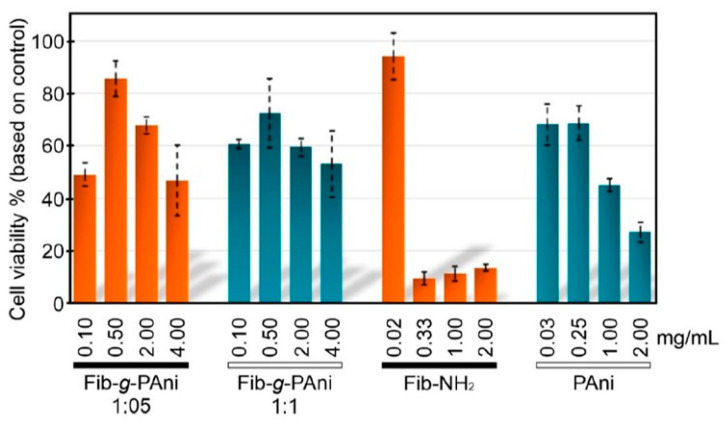
Cell viability (%) of NIH/3T3 fibroblast cells after exposure to various concentrations of Fib-g-PAni (1:05 and 1:1 mass ratios), Fib-NH₂, and polyaniline (PAni). The results demonstrate dose-dependent cytotoxicity, with Fib-g-PAni showing higher cell viability compared to pure PAni at similar concentrations, indicating improved biocompatibility due to the incorporation of fibroin. Reproduced and adapted with permission from [82].

In 2022, Flores-Vela et al. [82] reported the development of fibroin-g-polyaniline (Fib-g-PAni) copolymers synthesized through a one-pot method combining Fischer esterification and oxidative polymerization. The hybrid material, synthesized by grafting polyaniline chains onto silk fibroin, was designed to combine the biocompatibility and biodegradability of fibroin with the electroactive properties of polyaniline. The synthesis involved two key steps. As shown in Figure 4, initially, 3-aminobenzoic acid was esterified with fibroin to produce Fib-NH_2_, which served as a substrate for further functionalization. Subsequently, polyaniline chains were grafted onto the Fib-NH_2_ via oxidative polymerization. This process allowed for the production of Fib-g-PAni copolymers with varying mass ratios of fibroin to polyaniline, enabling control over material properties. The characterization of the hybrid material was carried out using a combination of ^1^H-NMR and FTIR spectroscopies, confirming successful grafting of polyaniline onto fibroin. Thermal stability was evaluated through thermogravimetric analysis, and morphological changes were studied using scanning electron microscopy. The results revealed significant structural transformations, with the copolymers exhibiting particulate and globular morphologies. The hybrid materials demonstrated electroactivity, with cyclic voltammetry tests confirming the presence of oxidation and reduction peaks typical of polyaniline. Cytotoxicity assays of the Fib-g-PAni copolymers using NIH/3T3 fibroblast cells showed that the copolymers exhibited low toxicity at certain concentrations, making them suitable for use in biocompatible platforms (Figure 5). The electroactive properties of the materials suggest their potential in developing electrochemical sensors that can interface with living tissues. Additionally, the combination of fibroin’s biocompatibility with polyaniline’s conductivity positions these materials as promising candidates for applications in tissue engineering and bioelectronics.

Kuang et al. [83] developed a silk fibroin-polyvinyl pyrrolidone (PVP-SF) interpenetrating polymer network (IPN) hydrogel designed to enhance the mechanical and optical properties of silk fibroin. The polymerization process relied on an enzymatic free-radical polymerization mechanism. In this process, n-vinyl-2-pyrrolidone (NVP) served as the monomer, and horseradish peroxidase (HRP) acted as the enzymatic catalyst. Hydrogen peroxide (H_2_O_2_) was used as the oxidizing agent to initiate polymerization. The resulting IPN hydrogels exhibited significantly improved properties compared to pure silk fibroin hydrogels. These showed superior light transmittance, achieving up to 97% transparency, and enhanced mechanical strength, with the ability to withstand up to 70% strain without rupture and resilience rates of up to 95%. These features make the IPN hydrogels highly suitable for advanced biomedical applications. The study emphasized potential applications of these IPN hydrogels in areas such as contact lenses and artificial vitreous materials, where high transparency, biocompatibility, and mechanical strength are critical.

**Figure 6:**
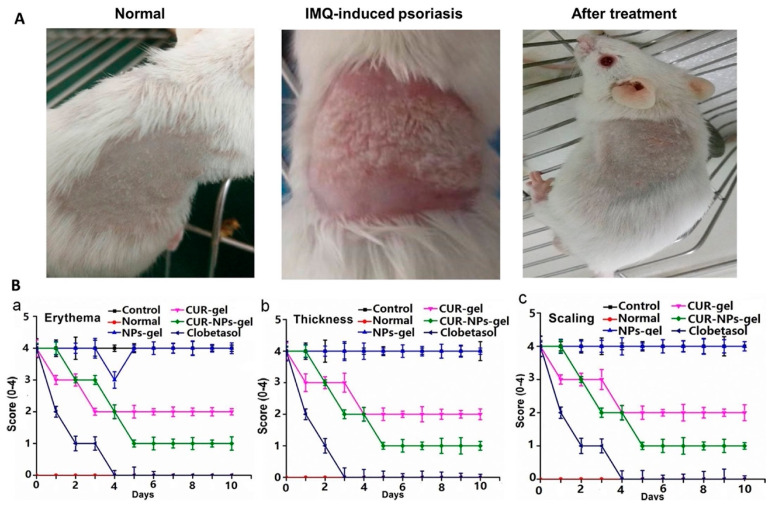
Preclinical evaluation of different therapeutic systems applied to control three main phenotypic psoriatic activities (erythema, thickness, and scaling) in an imiquimod-induced psoriatic mouse model. Different treatment and control groups were constituted as follows: Group 1 (Normal untreated mice); Group 2 (IMQ-induced psoriatic mice); Group 3 (Blank NPs-gel treatment); Group 4 (CUR-gel treatment); Group 5 (CUR-NPs-gel treatment); Group 6 (Clobetasol treatment as the positive control group). (**A**) Representative images of skin conditions: Normal skin, IMQ-induced psoriatic skin, and skin after treatment with various formulations. (**B**) PASI scoring of psoriatic skin over 10 days of treatment: (**a**) Erythema scores comparing control, normal, CUR-gel, CUR-NPs-gel, and clobetasol treatments; (**b**) Thickness scores for the same treatment groups; (**c**) Scaling scores across treatment groups. Data are expressed as Mean ± SD (n = 12) Reproduced with permission [89].

Psoriasis is a cutaneous inflammation process that is characterized by abnormal epidermal tissue proliferation. Mao and collaborators [89] designed a nanoparticle system with improved skin-permeating properties aiming to deliver curcumin to the deeper skin’s layers [89]. Seeking to improve the retention time of curcumin nanoparticles in the skin, the authors prepared a hydrogel-based matrix based on silk fibroin. The in vivo evaluation of the system was conducted in an imiquimod-induced psoriatic mouse model. The IMQ (imiquimod) cream was applied to the mice’s skin for 8 days consecutively. The drug clobetasol was used as a positive treatment control. The preclinical results are shown in Figure 6, and the data revealed that the proposed system could significantly reduce psoriatic phenotypes (erythema, scaling, and thickening) compared to control groups, showing relevant anti-psoriatic activity. The authors attributed the results to the capacity of the system to reach deeper skin layers and curcumin being retained in the CUR-NPs-SF hydrogel for a longer period.

In another study, Xing et al. [84] investigated grafting 2-diethylaminoethyl methacrylate (DEAEMA) onto silk fibroin, with optimization of parameters such as monomer concentration, reaction time, temperature, and catalyst ratios for maximum efficacy in generating fibroin-hybrid materials. This study highlighted DEAEMAs unique properties and focused on achieving not only antibacterial properties but also greater control over mechanical and chemical modifications. The hybrid material showed high grafting efficiency and robust washing resistance, with a focus on broader applications in textile engineering, material science, and potential biomaterial innovations.

**Figure 7:**
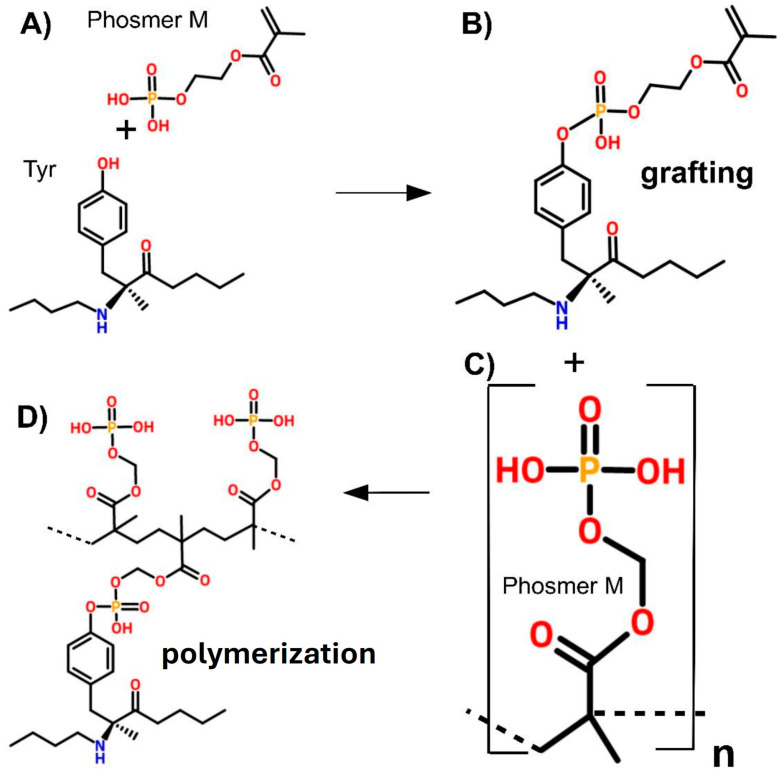
Scheme for the synthesis of the hybrid system. (**A**) Phosmer M reacts with silk fibroin, with tyrosine serving as the preferential reaction site; (**B**) grafting of a Phosmer M molecule onto the tyrosine side chain of silk fibroin; (**C**) and (**D**) subsequent polymerization. Reproduced and adapted with permission from supplementary material in [85].

Di Foggia et al. [85] proposed the functionalization of protein fibers, including silk fibroin (from bombyx mori and Antheraea pernyi) and wool keratin, by grafting phosphorylated methacrylate monomers (phosmers). These modifications were designed to enhance bioactivity and enable biomedical applications such as bone tissue engineering. Silk fibroin was grafted with two commercial phosphorylated methacrylate monomers, phosmer CL and phosmer M. The reaction was conducted at 80°C in an acidic environment using ammonium persulfate as an initiator. The degree of grafting was adjusted by varying the monomer concentration and reaction time, resulting in fabrics with different weight gains. Figure 7 summarizes the synthetic route used by the authors for the fibroin hybrid. It illustrates the process used to functionalize protein fibers, such as silk fibroin or keratin, with phosphorylated methacrylate monomers (phosmers). Initially (A), phosmer M reacted with the tyrosine residues of the protein, where the hydroxyl group served as the reactive site. In the grafting step (B), covalent bonds formed between phosmer M and the protein backbone, anchoring the monomers. Polymerization (C) was then initiated, with methacrylate groups reacting to form brush-like polymer chains extending from the protein surface. The final hybrid material (D) consisted of a protein backbone functionalized with phosphate-rich polymer chains, providing bioactive properties. The grafted fibers were characterized using Raman and infrared spectroscopies, which identified the phosphorylation of tyrosine and serine residues and confirmed the successful incorporation of phosphate groups into the fibers. The structural integrity of the proteins was largely maintained, with only minor changes observed in their secondary structures. β-sheet regions dominated after grafting, while amorphous regions showed some reactivity. The applications of these grafted fibers were demonstrated for the development of bioactive materials for bone regeneration. When immersed in simulated body fluid (SBF), the modified material demonstrated nucleation of carbonated calcium phosphate phases, indicating strong bioactivity. The phosphate groups, and to some extent sulfate/sulfonate groups, acted as nucleation sites for apatite formation, which is critical for bone tissue engineering. The grafted wool samples exhibited additional contributions from sulfur-containing groups, further enhancing their potential for mineralization. This work highlighted the potential of phosphorylated methacrylate-grafted protein fibers in creating bioactive materials for biomedical devices and tissue engineering applications, particularly for bone regeneration. The study suggested further research into long-term immersion effects and cellular interactions to expand the understanding of these materials’ biomedical potential.

Niu et al. [86] grafted a low molecular weight polyethyleneimine (LMW-PEI) onto the side chains in SF. The focus of this work was the preparation of structurally modified and cationized silk fibroin (CSF), capable of packaging plasmid DNA (pDNA) for applications in gene therapy. The polymerization does not occur on the surface of the protein but in the structure through the linked entities and reactive groups present in the protein through the grafting-to approach. PEI was linked to BSF, resulting in hybrid CSF-PEI, which was loaded with pDNA for the transfection of human lung adenocarcinoma cells (A549 model) and human embryonic lung fibroblast cells (WI-38 model) in order to modulate the gene expression on target cells. The complex CSF/pDNA was able to significantly inhibit A549 cell proliferation; however, the hybrid system displayed little effectiveness and cytotoxicity against WI-38 cells, demonstrating that the hybrid system has potential for gene therapy applications against lung cancer. 

Recently in 2024, Viola et al. [88] studied hybrid materials based on silk fibroin hydrogels reinforced with melt electro-written fibers of a methacrylated polymer (pMHMGCL). The hybrid system was synthesized by covalently bonding methacrylated silk fibroin (silkMA) to the fibers via photopolymerization. The hybrid material exhibited enhanced mechanical properties, including a threefold increase in compression resistance and a 40–55% increase in tensile strength compared to systems without covalent interactions. Articular cartilage progenitor cells embedded in the silkMA hydrogels produced a cartilage-like matrix after 28 days of in vitro culture, demonstrating the material’s applicability in cartilage tissue engineering. This approach highlighted the capability of hybrid silk fibroin systems to fulfill the mechanical and biological requirements of tissue engineering applications, particularly for load-bearing tissues like cartilage.

The exploration of hybrid silk fibroin-polymer systems has demonstrated the remarkable potential of combining natural proteins with synthetic polymers to create materials with tailored properties and broadened applications. Through chemical modifications, such as grafting with hydrophilic, zwitterionic, phosphorylated, or electroactive polymers, fibroin’s intrinsic biocompatibility and biodegradability can be complemented by functionalities such as mechanical strength, thermal stability, conductivity, and environmental responsiveness. The synthesis strategies, ranging from ATRP to oxidative polymerization, have shown that precise control over polymer grafting is key to tailoring the hybrid materials for specific needs. Characterization techniques like FTIR, NMR, thermal analysis, and microscopy have confirmed the successful incorporation of polymers while maintaining fibroin’s structural integrity. These modifications address fibroin’s natural limitations, such as enzymatic degradation and low stability, by introducing properties like improved bioactivity, resistance to biofouling, and tunable mechanical and surface characteristics. Applications of these hybrid systems span various biomedical fields, including drug delivery, tissue engineering, wound healing, and bioelectronics. The ability to design fibroin hybrids with controlled drug release profiles, enhanced cell interactions, and even electroactive capabilities underscores their versatility. For instance, fibroin-grafted polyaniline systems show promise in developing electrochemical sensors and conductive scaffolds, while phosphorylated fibroin systems offer solutions for bone tissue engineering by promoting mineralization.

These studies clearly demonstrate that fibroin-based hybrid systems exemplify the synergy between natural and synthetic materials while serving as a versatile platform for tackling complex biomedical challenges. The ability to fine-tune their properties through controlled synthesis highlights their potential as next-generation biomaterials, capable of bridging the gap between structural functionality and biological compatibility. This field continues to pave the way for innovative solutions in healthcare and material science. Gene therapy is based on the use of exogenous nucleic acids (genes, genetic segments, RNA, DNA, and others) with the ability to modify patients’ genes and treat or extinguish diseases [90,91]. This therapy can act through different mechanisms, for example, replacement of the disease-causing gene by a healthy copy, inactivation of the disease-causing gene, or even by introducing a new or modified gene to help in the treatment of a disease [90]. Due to its great versatility and application potential, this therapy has received much attention in recent decades for the treatment of diseases caused by genetic abnormalities [92].

Despite the great potential of gene therapy, “naked” therapeutic nucleic acids do not present efficient internalization in target sites, since they are susceptible to nuclease attack, uptake by phagocytes, renal clearance, and stimulation of the immune response, factors that make clinical applications of these molecules unfeasible [93]. To achieve the expected action and overcome such limitations, it is essential to use efficient carrier systems (vectors), which can be viral or non-viral [91]. Taking advantage of the properties and nature of viruses, viral vectors were the first ones to be proposed for gene therapy. However, these systems raise concerns mainly related to immune response [5]. Alternatively, several studies have been carried out to develop nanostructured systems capable of achieving better immunological and toxicological responses and, therefore, safer [94,95,96]. Silk protein has gained great prominence due to its biocompatibility, DNase resistance, and especially high transfection efficiency [96]. These properties associated with the use of modern synthetic strategies have enabled the development of smart fibroin-based nanostructured systems. These new systems can be designed to have well-defined sizes, high stability, and efficiency in targeting, which makes them very promising for application as non-viral vectors [5,97,98].

SF methacrylates (SilMA) have attracted the attention of many researchers in tissue engineering and, especially for wound healing, proliferation and cell growth stimulation. Gong and collaborators [99], in 2019, studied the encapsulation of octreotide acetate in fibroin microspheres, a peptide that has been used for the treatment of gastrointestinal tumors and acromegaly. The main motivation of this work was due to incomplete release and acylation reactions of the encapsulated octreotide in available pharmaceutical formulations based on PLGA microspheres (i.e., Sandostatin^®^) [99]. Furthermore, acidic degradation product-induced inflammation has resulted in major challenges hampering widespread clinical applications of this delivery system.

**Figure 8:**
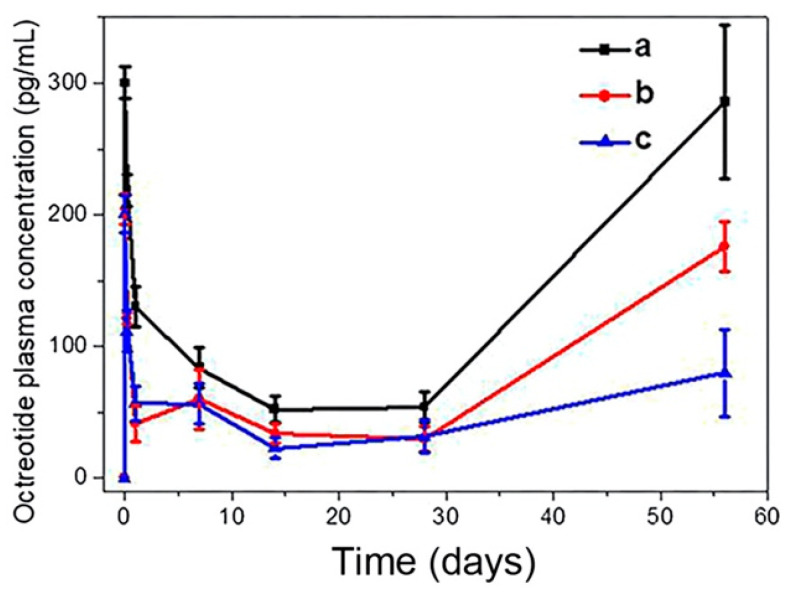
Octreotide concentration profile in rat plasma after intramuscular injection. (**a**) elevated dose group (8 mg/kg), (**b**) reduced dose group (2 mg/kg), (**c**) blank octreotide group at reduced dose. Reproduced with permission [99].

The authors developed a new method to fabricate microspheres using fibroin and polyethylene glycol (high molecular weight PEG) through an assisted emulsification method. They studied the in vivo pharmacokinetics behavior of the system, which was intramuscularly injected in healthy mice. The results revealed a sustained release profile for 102 days. The authors observed an important increase in plasma concentration of octreotide, which was attributed to slow degradation of the fibroin microspheres matrix, promoting a linear sustained release profile (Figure 8).

## 4. Inorganic-Fibroin Hybrid Systems

The development of fibroin-polymer hybrid systems has demonstrated promising advances for their applications in the biomedical field. In a similar vein, other hybrid systems based on fibroin have been explored, which include the integration of inorganic components, such as silica and hydroxyapatite [22,23,24,100]. In the biomedical field, systems based on silica and fibroin offer a unique combination of thermal stability and mechanical strength of silica with the biological properties of fibroin, resulting in novel materials [16,24,101]. These systems could be utilized in tissue engineering, where silica contributes to structure and rigidity, while fibroin promotes cell adhesion and growth, and for controlled drug release, where the hybrid matrix can control release kinetics and increase therapeutic efficacy [16,100,101,102,103].

Silk-based scaffolds, in the last decade, have been the target of many studies, in particular, related to bone tissue regeneration, since mesoporous bioactive glass (MBG)-silk scaffolds have shown in vitro bioactivity and in vivo osteogenic properties. Such characteristics can be related to a favorable environment for cell attachment, proliferation, and differentiation, making the silk-silica hybrid system a potential substitute for treating local osteoporotic defects [104,105,106]. Table 3 provides an overview of hybrid inorganic systems, highlighting their properties, applications in biomedical engineering, characterization techniques, and biomedical assays.

Saleem et al. [22] investigated SF membranes combined with hydroxyapatite (HA) to evaluate their physico-chemical properties and biomedical applications, specifically in bone regeneration and wound healing. Characterization revealed that HA crystals were uniformly deposited on the SF membrane, with SEM and EDS confirming the presence of calcium phosphate structures. FTIR analysis indicated the disappearance of fibroin-specific bands, likely masked by HA coating. Mechanical tests showed reduced strength and energy expenditure for SF-HA membranes compared to SF while maintaining sufficient elasticity, especially in wet conditions, ideal for in vivo use. Biocompatibility assays demonstrated that SF-HA membranes exhibited no cytotoxic, genotoxic, or mutagenic effects, unlike unmodified SF membranes, which showed toxicity and compromised cell viability. The hybrid system supported cell adhesion and proliferation, essential for biomedical applications. The inclusion of HA enhanced osteoconductivity, making these membranes suitable for bone regeneration and tissue engineering. Overall, the findings highlighted SF-HA composites as promising materials for clinical use in wound healing and bone repair applications.

Maleki et al. [24] developed a silica-silk fibroin hybrid bioaerogel using sol-gel and freeze-casting, resulting in a porous, honeycomb-structured scaffold with excellent mechanical properties. The synthesis involved a one-pot aqueous sol-gel reaction using tetraethyl orthosilicate and silk fibroin, followed by unidirectional freeze-casting and supercritical drying. The material supported osteoblast attachment and promoted bone regeneration in vitro and in vivo, demonstrating its potential for bone tissue engineering. The hybrid scaffold was non-toxic and biocompatible, making it a promising candidate for clinical applications in bone repair.

In 2018, Pereira et al. [101] developed a hybrid system constituted of silk fibroin and silica aiming to enhance the mechanical properties and host viable MC3T3 pre-osteoblast cells. The authors obtained ordered mesoporous bioglasses (MBGs) fibroin/silica hybrid implants through sol-gel reactions, and the implant resulted in a hybrid system with superior characteristics. Although MBGs display high surface area and pore volume, the material exhibited high in vivo degradation rates and significant surface instability, compromising the mechanical stability and the cytocompatibility of the material. The presence of fibroin in the hybrid conferred a relevant improvement over these drawbacks, allowing the design of materials with tunable morphology and mesoporosity [101].

**Figure 9:**
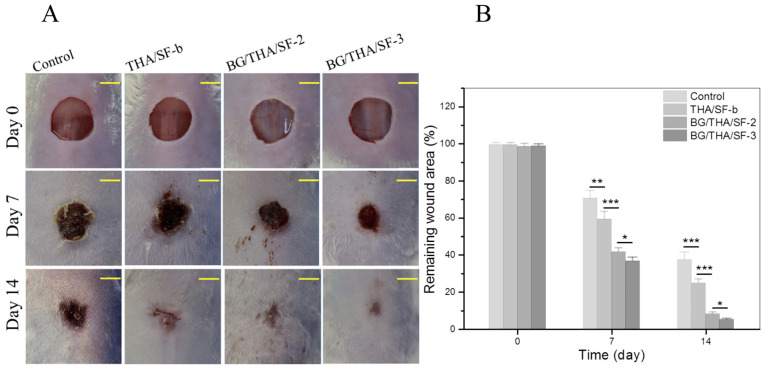
Assessment of wound healing: (**A**) Illustrative images of the wound area versus various gel groups and (**B**) calculated remaining wound area for different groups on days 0, 7, and 14, respectively (n = 4) (* *p* < 0.05; ** *p* < 0.01; *** *p* < 0.001). Reproduced with permission [107].

More recently, Yu and collaborators [107] prepared a hybrid system combining thiolated hyaluronic acid (THA), SF, and bioactive glass (BG) nanoparticles. The resulting material displayed enhanced properties related to strength, stability, elasticity, and other functionalities compared to the single-network-based THA gel only [107]. In terms of wound healing evaluation, the authors used Balb/c mice in the experiments that had a circular, full-thickness wound area fully covered with different preparations based on the prepared hybrid system: (i) THA/SF-b; (ii) BG/THA/SF-2; or (iii) BG/THA/SF-3, which were prepared with different amounts of THA, silk fibroin, and bioglass nanoparticles. A control group of mice had wounds left uncovered without any gels. The control group and assay groups were photographed on days 0, 7, and 14, and the results are shown in Figure 9. The data indicated that THA/SF-b, BG/THA/SF-2, and BG/THA/SF-3 gels had a significant impact on wound healing, as evidenced by the notable decrease in remaining wound areas over time. For example, on day 7, the remaining wound areas in the BG/THA/SF-2 and BG/THA/SF-3 groups were considerably smaller compared to the control or THA/SF-b groups. This suggests a rapid response of BG/THA/SF-2 and BG/THA/SF-3 gels in promoting wound healing. After 14 days of treatment, the wounds treated with the BG/THA/SF-2 or BG/THA/SF-3 gels were almost completely healed, while the wounds in the other two groups were still present.

Quantitative analysis of the remaining wound areas demonstrated significant differences between the groups on days 7 and 14, with emphasis on the BG/THA/SF-3 group, which presented a significantly smaller remaining wound area compared to the BG/THA/SF-2. This indicates that the BG/THA/SF-3 gel may have even greater potential in promoting wound healing than BG/THA/SF-2. These results suggest that BG/THA/SF gels have the ability to significantly accelerate wound closure, making them promising material for clinical applications in the area of wound healing.

Zhao et al. [108] synthesized a novel hybrid scaffold composed of silk fibroin, hydroxyapatite, and naringin (NG) using a salt-leaching process. The hybrid scaffold exhibited favorable biodegradability, porosity, and mechanical properties, making it ideal for bone tissue engineering applications. The incorporation of naringin enhanced osteogenesis and angiogenesis, as demonstrated by in vitro assays with human umbilical cord mesenchymal stem cells and in vivo studies in rabbit femur defect models. This scaffold provided superior bone regeneration and vascularization compared to controls, showing promise as a clinical solution for bone defect repair.

Coelho et al. [109] prepared silk fibroin membranes functionalized with hydroxyapatite (HA) to enhance their suitability in bone tissue engineering and wound healing. The biomedical potential of these membranes was thoroughly evaluated through in vitro assays using CHO-K1 cells. The SF-HA membranes demonstrated excellent biocompatibility, showing no cytotoxicity, genotoxicity, or mutagenicity, which were limitations observed in unmodified SF membranes. The hybrid membranes supported cell adhesion, proliferation, and viability, crucial factors for effective tissue regeneration. These results underscore their capability to serve as scaffolds that facilitate cell colonization and promote tissue repair. In addition to their in vitro performance, the SF-HA membranes exhibited significant potential for in vivo applications, particularly in bone regeneration. The osteoconductive nature of HA enabled the hybrid material to guide bone growth and mineral deposition, essential for healing critical bone defects. The improved mechanical and biological properties of the SF-HA membranes make them a promising solution for clinical applications in bone defect repair, wound healing, and broader regenerative medicine strategies.

The development of hybrid inorganic-fibroin systems has revealed significant potential in biomedical applications, particularly in bone regeneration, wound healing, and tissue engineering. The integration of inorganic components such as silica and hydroxyapatite (HA) into silk fibroin matrices has demonstrated the ability to combine the mechanical strength and thermal stability of these materials with the biocompatibility and cell adhesion properties of fibroin. These hybrid systems provide a multifaceted approach in overcoming challenges for creating functional and biologically active scaffolds. Overall, inorganic-fibroin hybrid systems represent a versatile and promising class of materials, bridging the gap between mechanical functionality and biological activity. Their tunable properties and demonstrated efficacy across various biomedical assays highlight their potential for advancing clinical applications in tissue repair and regenerative medicine. Continued exploration of these systems may lead to novel solutions for unmet medical needs, driving innovation in biomaterials science.

## 5. Conclusions and Future Outlook

There has been a recent surge in interest and research surrounding fibroin-based hybrid materials, especially for biomedical applications. This emerging and promising area of study presents vast opportunities for further investigation and development. This review offers an overview of the current state of the art, in which we have compiled and analyzed some of the most relevant advances in the field. It offers valuable insights into the potential and challenges of fibroin hybrids, establishing a solid foundation for future research in this rapidly evolving field. As discussed above, fibroin offers important properties, including biocompatibility and a low immunogenicity profile, making it a most promising material for biomedical applications. The presence of reactive groups on the fibroin’s protein structure has offered the possibility of chemical modification that can help further advance its scope. Such modifications can also help facilitate the fabrication of hybrid materials with additional virtues such as responsivity to external or endogenous stimuli. The fabrication of such smart hybrids could lead to systems that would respond to stimuli activated through a change in the chemical or physical environment, temperature, enzymatic action, pH, applied magnetic field, or ultrasounds. The development of such materials will help enhance the intrinsic properties of the hybrids that can amplify their biomedical applications.

The development of fibroin-polymer hybrid systems has demonstrated the significance of such systems. However, to fully exploit their potential, it is necessary to develop an understanding of the structure-property relationships. Additionally, we need to elaborate on i) the production cost of polymer-hybrid systems, which presents a significant barrier to advancing to clinical trials, and ii) predicting and examining biological interactions with cells during the early design stage, which remains a major challenge. It is essential that the scientific community address significant issues in designing tailored hybrid systems, which include selection of chemical functionalities for bioconjugation, development of effective conjugation schemes, and optimization of polymer compositions and architectures. An evaluation of the desired physicochemical properties in the resulting systems is also a critical and complex task. There has been good progress in the synthesis of hybrid systems and an evaluation of their potential in vitro and preclinical applications. However, a detailed comparative analysis of fibroin-hybrid systems to other relevant systems, including biocompatibility and low-immunogenicity profiles, will help expand the scope of fibroin-based materials. There is a critical need to evaluate their in vivo stability, enhancing drug payload capacity, endosomal escape ability for cancer drug delivery, reduced immune response (especially those related to the non-specific antibody production), and effective stimulus responsivity using external cues (lasers, magnetic field, or heat). More data from in vitro and preclinical assays need to be acquired to elucidate and consolidate unique characteristics of fibroin-hybrid systems compared to non-hybrid systems that have been well studied.

## Figures and Tables

**Table 2:** Summary of silk fibroin-polymer hybrid systems, elaboration of the enhanced properties, potential biomedical applications, employed assays, and physicochemical characterization methods.

Hybrid System	Properties	Potential Applications	Characterization Methods	Biomedical Assays/Next Steps	Reference
Silk fibroin-graft PMMA and PTBSiMA	Improved thermal stability, antifouling properties	Marine coatings, potential biomedical surfaces	FTIR, Thermal Analysis, SEM	Antifouling tests in aqueous environments	[52]
Silk fibroin-g-PDMAEMA	Antibacterial properties, controlled grafting, and durability	Antibacterial textiles and biomedical surfaces	FTIR, XPS, SEM	Antibacterial activity against *S. aureus* and *E. coli*	[80]
Silk fibroin-g-PNIPAM	Thermosensitive response, controlled drug release	Smart drug delivery systems	FTIR, DSC, TGA, SEM	Drug release studies with 5-FU	[81]
Silk fibroin-g-PANI	Electroactive properties, low cytotoxicity, good biocompatibility	Biocompatible sensors, electrochemical devices	^1^H-NMR, FTIR, Cyclic Voltammetry, SEM	Cytotoxicity assays with NIH/3T3 cells	[82]
Silk fibroin/Polyvinyl Pyrrolidone (PVP)	High transparency, enhanced mechanical strength, elasticity	Contact lenses, artificial vitreous materials	FTIR, SEM, XRD, Transmittance	Enzymatic Degradation of Hydrogels in vitro	[83]
Silk fibroin-g-DEAEMA	Antibacterial properties, good washing durability	Antibacterial clothing and textiles	FTIR, Raman, Washing Durability	Antibacterial assays, washing durability tests	[84]
Silk fibroin-graft phosphorylated methacrylates	Bioactivity, calcium phosphate nucleation	Bone tissue engineering, flame-retardant applications	FTIR, Raman, XRD, SEM	Bioactivity assays for calcium phosphate deposition	[85]
Polyethyleneimine-modified silk fibroin	Cationization, enhanced DNA packaging, higher transfection efficiency	Gene delivery systems for cancer therapy	FTIR, Gel Electrophoresis, Cytotoxicity Assays	Gene transfection efficiency tests in A549 cells	[86]
Silk fibroin-graft hydrophilic/zwitterionic polymers	Improved hydrophilicity, reduced protein and cell attachment, tunable beta-sheet content	Drug delivery implants, anti-biofouling surfaces, tissue engineering scaffolds	ATR-FTIR, AFM, Contact Angle Measurements, Degradation Studies	Bovine serum albumin (BSA) adhesion studies, human mesenchymal stem cell (hMSC) attachment assays	[87]
SilkMA + pMHMGC	Enhanced mechanical strength, elasticity	Cartilage tissue engineering	Compression testing, FTIR, SEM	Cartilage-like matrix production in vitro	[88]

FTIR: Fourier transform infrared spectroscopy XPS: x-ray photoelectron spectroscopy, TGA: thermogravimetric analysis, DSC: differential scanning calorimetry, ^1^H-NMR: proton nuclear magnetic resonance spectroscopy SEM: scanning electron microscopy, XRD: x-ray diffraction, UV-Vis: ultraviolet-visible spectroscopy, ATR-FTIR: attenuated total reflection Fourier transform infrared spectroscopy AFM: atomic force microscopy, GPC: gel permeation chromatography, BSA: bovine serum albumin, MSC: mesenchymal stem cells, 5-FU: 5-fluorouracil, DNA: deoxyribonucleic acid, DEAEMA: 2-diethylaminoethyl methacrylate, PANI: polyaniline, PDMAEMA: polydimethylaminoethyl methacrylate, PNIPAM: poly(N-isopropylacrylamide), PTBSiMA: poly(tributylsilyl methacrylate), pMHMGC: methacrylated polymers, MA: methacrylate, PMMA: poly(methyl methacrylate).

**Table 3:** Summary of silk fibroin-inorganic hybrid systems, elaboration of the enhanced properties, potential biomedical applications, employed assays, and physicochemical characterization methods.

Hybrid System	Properties	Potential Applications	Characterization Methods	Biomedical Assays/Next Steps	Reference
SF/HA	Enhanced mechanical and osteogenic properties	Bone regeneration, tissue scaffolds	SEM, FTIR, Mechanical Testing	Osteoblast adhesion, proliferation	[22]
SF/SiO_2_ Aerogel	High porosity, hierarchical structure	Bone tissue engineering	XRD, BET, Mechanical Testing	Osteoconduction assays, in vivo implant tests	[24]
SF/Silica Hybrid	Enhanced bioactivity, porosity	Drug delivery, bone tissue engineering	Sol-gel characterization	Cytotoxicity Pre-osteoblast proliferation and viability	[101]
THA/SF/BG Hydrogel	Strength, elasticity, wound healing	Wound healing	Rheology, Cell Migration Assays	In vivo wound closure analysis	[107]
NG/SF/HA Scaffold	Porosity control, angiogenic properties	Bone defect repair	SEM, FTIR, ALP Assay, ARS	Angiogenesis, osteogenic differentiation	[108]
SF/hydroxyapatite composite	biocompatibility, mechanical strength, hydrophilicity, cytotoxicity-free (with HA), genotoxicity-free	Bone regeneration, wound healing	SEM, EDS, FTIR, mechanical testing, contact angle measurement	Cytotoxicity (XTT assay), genotoxicity, mutagenicity (micronucleus test)	[109]

SEM: scanning electron microscopy, FTIR: Fourier transform infrared spectroscopy, XRD: X-Ray diffraction. BET: Brunauer-Emmett-Teller analysis, ALP: alkaline phosphatase assay, ARS: alizarin red S staining, EDS: energy dispersive spectroscopy, THA: thiolated hyaluronic acid, SF: silk fibroin, BG: bioactive glass, NG: naringin, HA: hydroxyapatite.

## Data Availability

The data presented in this study are available in this article.
